# Supplementary material for: Complexome profiling of the Chlamydomonas psb28 mutant reveals TEF5 as an early PSII assembly factor
Source: Plant Cell. 2025 Mar 17;37(6):koaf055. doi: 10.1093/plcell/koaf055 (PMC12164590; doi:10.1093/plcell/koaf055)
Supplement: koaf055_Supplementary_Data [file koaf055_supplementary_data.zip › psb28_interactive.zip!/plots/intensity/qualityPlots/peptides/_Cre14.g617826.t1.1.html]

### Cre14.g617826.t1.1

Trivial name:   
SeqCoverage [median]: 26.90  
Mass: 23.37 kDa  
  
 

| Sequence | MissedCleavage | Mass [Da] | wtIntensity | muIntensity | Score |
| --- | --- | --- | --- | --- | --- |
| IYGPVVHR | 0 | 939.53 | 0.00 | 2662.44 | 129.96 |
| QAQLLLGIIGK | 0 | 1152.72 | 5419.01 | 0.00 | 178.94 |
| SSEAEYGVDTPVPASALK | 0 | 1819.88 | 23113.30 | 22140.69 | 281.26 |
| TKSSEAEYGVDTPVPASALK | 1 | 2049.03 | 5936.93 | 6170.75 | 95.96 |
| VFSGAFNTPSSTTAR | 0 | 1541.75 | 21907.07 | 10865.05 | 234.79 |
| VFSGAFNTPSSTTAREPYAK | 1 | 2130.04 | 10200.83 | 22281.45 | 143.46 |
|  |  |  | 66577.1 | 64120.4 |  |
